# A Unique Combination of Male Germ Cell miRNAs Coordinates Gonocyte Differentiation

**DOI:** 10.1371/journal.pone.0035553

**Published:** 2012-04-20

**Authors:** Skye C. McIver, Simone J. Stanger, Danielle M. Santarelli, Shaun D. Roman, Brett Nixon, Eileen A. McLaughlin

**Affiliations:** 1 ARC Centre of Excellence in Biotechnology and Development, Discipline of Biological Sciences, School of Environmental and Life Sciences, University of Newcastle, Callaghan, New South Wales, Australia; 2 School of Biomedical Sciences and Pharmacy, University of Newcastle, Callaghan, New South Wales, Australia; The Institute of Cancer Research, London, United Kingdom

## Abstract

The last 100 years have seen a concerning decline in male reproductive health associated with decreased sperm production, sperm function and male fertility. Concomitantly, the incidence of defects in reproductive development, such as undescended testes, hypospadias and testicular cancer has increased. Indeed testicular cancer is now recognised as the most common malignancy in young men. Such cancers develop from the pre-invasive lesion Carcinoma *in Situ* (CIS), a dysfunctional precursor germ cell or gonocyte which has failed to successfully differentiate into a spermatogonium. It is therefore essential to understand the cellular transition from gonocytes to spermatogonia, in order to gain a better understanding of the aetiology of testicular germ cell tumours. MicroRNA (miRNA) are important regulators of gene expression in differentiation and development and thus highly likely to play a role in the differentiation of gonocytes. In this study we have examined the miRNA profiles of highly enriched populations of gonocytes and spermatogonia, using microarray technology. We identified seven differentially expressed miRNAs between gonocytes and spermatogonia (down-regulated: miR-293, 291a-5p, 290-5p and 294*, up-regulated: miR-136, 743a and 463*). Target prediction software identified many potential targets of several differentially expressed miRNA implicated in germ cell development, including members of the PTEN, and Wnt signalling pathways. These targets converge on the key downstream cell cycle regulator Cyclin D1, indicating that a unique combination of male germ cell miRNAs coordinate the differentiation and maintenance of pluripotency in germ cells.

## Introduction

Over the last 100 years there has been a substantial increase in diseases of the male reproductive system including developmental abnormalities, poor semen quality and testicular cancer, especially in developed countries [1]–[3]. The rising incidence of type II testicular cancer is highly correlated with infertility as well as more overt problems of reproductive health suggesting that it is an indicator of a broader problem with the general reproductive health of the population [4]. There is concern that exposure to environmental toxicants *in utero*, especially endocrine disruptors, augments a genetic predisposition for testicular germ cell tumours such as defects in kit signalling (KITLG itself and SPRY an inhibitor of kit stimulated MAPK signalling), apoptosis (BAK), sex determination (DMRT1) and telomere regulation (TERT) [5], [6]. This is particularly pertinent given that the gonocyte-to-spermatogonia transition occurs in late gestation in humans [7] and that Carcinoma *in Situ* (CIS) cells have previously been identified as arising from arrested/dysfunctional gonocytes [8]. Taken together these findings suggest that the risk for testicular cancer must therefore be established *in utero*.

MicroRNAs (miRNA) are short untranslated RNA molecules which bind to and post-transcriptionally regulate mRNA expression. Since their discovery in 1993, they have been found to be essential regulatory molecules in many developmental and cell signalling pathways [9]. Although some miRNA are ubiquitously expressed, the majority of miRNA species are expressed in a tissue specific manner [10], [11]. MicroRNA molecules are produced by an endonuclease Dicer, associate with a protein complex (RISC) in the cytoplasm, and target mRNA molecules by complementary base pairing, usually in the 3′UTR. Binding of the RISC complex to mRNA causes transcript degradation, mRNA sequestering or inhibition of translation [9]. In contrast, when the cell cycle is arrested, miRNA can promote the translation of mRNAs containing AU rich elements [12]. A study of 130 non-redundant proteins in Sertoli cell-specific Dicer knockout mice, demonstrated that 50 proteins were up-regulated and three proteins were down-regulated, indicating that miRNA are primarily involved in the inhibition of protein translation [13].

After primordial germ cells are specified in early embryogenesis they migrate through the hindgut to reach the genital ridges, where they are termed gonocytes. Within the gonads gonocytes undergo sex specific differentiation to become prospermatogonia [14], [15]. Shortly after birth in mice and late gestation in human, prospermatogia (gonocytes) migrate to the basement membrane of the seminiferous tubules and differentiate into spermatogonia [16]. When perturbed this can result in the specification of CIS cells instead of prospermatogonia [7], [8]. After puberty spermatogonial stem cells maintain an undifferentiated population while specifying some daughter cells to undergo the process of spermatogenesis to produce sperm. Differentiating spermatogonia undergo transiently amplify their numbers by mitosis before becoming type B spermatogonia which divide by meiosis to become spermatocytes. Spermatocytes divide again without replicating their DNA to become haploid spermatids, which differentiate in a process known as spermiogenesis to become mature spermatozoa [15].

Spermatogenesis involves many unique genes [17], the expression of which are translationally uncoupled from protein production in germ cell development, a process thought to be partially coordinated via miRNA suppression [13]. The regulation of genes involved in meiosis and spermatid differentiation by miRNA has been found to be essential for functional spermatogenesis [18]–[20]. Interestingly the miRNA testis expression profile is unique; for example, Linsen et al [11] found fewer miRNA in rat testis tissue than in the other tissues studied, yet 35 of these were specific to the testis. In a cloning study, Ro et al [21] identified 141 miRNA expressed in mouse testis, of which 35% were preferentially expressed in the testis and 5% were unique to the testis.

Aberrant expression of miRNA molecules and mutations in their recognition sites on mRNA are associated with infertility in men [22], [23]. Of particular note the oncogenic miR-17-92 as well as the oncogenic cluster linked to testicular cancer, miR-371-373, were down-regulated in infertile men. It is proposed that the lack of protection against apoptosis provided by these miRNAs caused developing germ cells to die before differentiating into spermatozoa [23]. Furthermore, studies in the mouse [24] and chicken [25] have demonstrated changes in miRNA expression after exposure to toxins. Oncogenic miRNAs including miR-17-20 tended to be up-regulated after xenobiotic exposure while tumour suppressor miRNAs such as let-7, tended to be down-regulated [24]. Therefore it is highly probable miRNA play a key role in the response to toxic challenge in many different organs of the body, and could in fact contribute to the development of testicular cancer following exposure to environmental toxicants *in utero*. In proliferating cells miRNA perform key regulatory roles by either promoting or inhibiting the cell cycle and safeguard organismal integrity by inducing apoptosis following DNA damage. For example, the well-known tumour suppressor p53, activates the miR-34 family, which in turn halts cell proliferation and arrests adhesion independent growth in cancer cell lines [26]. E2F1 which promotes cell cycle progression, while also inducing apoptosis in damaged cells, positively regulates miR-449a and 449b expression. miR-449a has been found in high levels in the normal testis but is absent in testicular tumours. Also miR449a/b promotes apoptosis in a p53 independent manner in tumour cell lines, indicating this miRNA family are also key regulators of apoptosis [27]. Type II testicular germ cell tumours develop from transformed gonocytes (CIS) which proliferate and become neoplastic after puberty [8], [28]. Expression profiling of miRNA in testicular germ cell tumours has identified several key miRNA molecules which allow germ cell tumours to bypass traditional mechanisms of cancer development [29]. Gillis et al [30] profiled the expression of type I, II and III testicular germ cell tumours and identified some miRNA species as tumour specific.

Studies on conditional germ cell Dicer knockout mice indicate that miRNA are essential for the formation and maintenance of spermatozoa, as without Dicer spermatogenesis stalled just before the elongating spermatid stage [18], [19]. In particular, miRNA regulation of protamination is essential for functional spermatogenesis [31], [32]. Importantly, the expression pattern of miRNAs changes in germ cells during development, indicating that miRNAs are likely to play a regulatory role in differentiation [33]–[35].

Despite their importance for spermatogenesis, global miRNA expression studies focussing on crucial stages of germ cell development have been limited, especially in isolated cells types [15]. The cloning study previously described [21] further examined the expression of 28 miRNA molecules they found to be testis specific in Sertoli cells and isolated germ cells at five different developmental stages; spermatogonia, pachytene spermatocyte, round spermatid, elongating spermatid and spermatozoa. Another study concentrated on enriched meiotic germ cells finding that these cells have the highest concentration of miRNA of the germ cells within the testis, but unfortunately this study suffered from problems with contamination [36]. More recently several additional novel miRNA species and splice variants have been identified within the testis, further indicating the testis has a unique miRNA expression profile [37], [38].

To date there has been no study on global expression of miRNA in isolated germ cells during the gonocyte to spermatogonial transition. This particular developmental stage is of interest given that the pre-invasive stage of testicular germ cell tumours, CIS, are derived from a population of dysfunctional gonocytes [8]. To address this we have examined the global change(s) in expression between enriched populations of murine gonocytes and spermatogonia. Only a few miRNA molecules were expressed at significantly different levels between gonocytes and spermatogonia, indicating that small changes in the miRNA expression between cell types can lead to significant changes in the proteomic profile of these cells. These miRNA molecules have the potential to regulate many signalling pathways though the regulation of their target mRNA molecules, such as key signalling molecules such as hormone receptors, second messengers such as AKT, ERK and MEK, as well as transcription factors including the SOX proteins. A number of potential targets of the differentially expressed miRNA molecules play a role in PTEN and WNT signalling pathways, both of which have been previously found to be important in spermatogenesis as well as tumour development.

## Materials and Methods

### Animals and reagents

#### Ethics Statement

The use of animals for this study was approved, and animals maintained and euthanised in accordance with directions prescribed by the University of Newcastle Animal Care and Ethics Committee (ACEC) (approval number A-2010-146).

All chemicals, and reagents were obtained from either Sigma (Sigma-Aldrich, St. Louis, MO, USA), Research Organics (Research Organics Inc., Cleveland, OH, USA), Promega (Promega, Madison, WI, USA), or Invitrogen (Invitrogen Corporation Carlsbad, CA, USA) unless otherwise stated. Antibodies were purchased from Abcam (Cambridge, MA, USA): anti-SOX2 (ab42635), anti-SOX11 (ab59776), anti-FZD4 (ab83042), anti-FZD7 (ab51049), anti-AKT1/2 (ab8933), anti-Cyclin D1 (ab16663), anti-UCHL1 (ab10404), anti-OCT3/4 (ab27985); Santa Cruz Biotechnology (Santa Cruz, CA, USA): anti-PTEN (sc7974), anti-SMAD4 (sc7154); Biocore (Sydney, NSW, Australia): anti-BMPR1a (ap2004b); Calbiochem (Merck KGaA, Darmstadt, Germany): anti-PLZF (op128); or Sigma: anti-ERK1/2 (M5670), anti-MEK1/2 (M5795) and anti-α-tubulin (T5168).

### Germ cell isolation and characterisation

**Figure 1:**
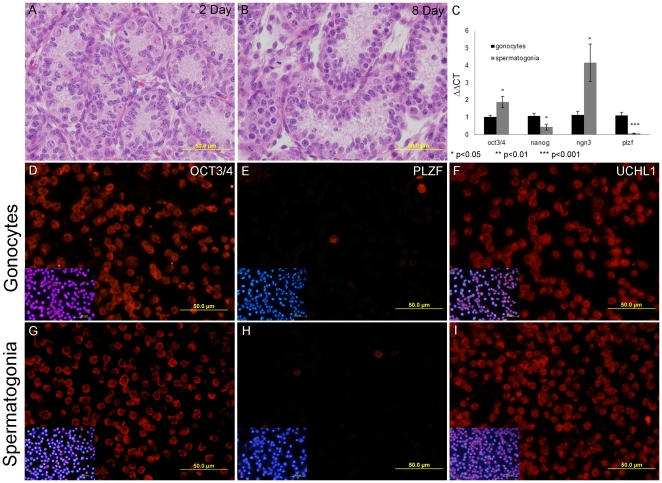
Characterisation of isolated gonocyte and spermatogonia cells. Haematoxylin and eosin stained two day old testis section (1A) demonstrates that gonocytes are found in the centre of the seminiferous tubules of mice. Gonocytes differentiate and migrate to the basement membrane of the seminiferous tubules where they become established in their niche and begin the process of spermatogenesis as seen in the eight day old testis section stained with haematoxylin and eosin (1B). C) Total RNA from isolated germ cell populations (three biological replicates) reverse transcribed and subjected to qPCR. It was found that the gene expression of the germ cell markers *Oct3/4* and *Ngn3* were significantly higher (p<0.05) in spermatogonia, twice and four times the amount of gonocytes respectively. The gene expression of the ES cell markers *Nanog* and PLZF was significantly lower (p<0.05 and 0.001) in spermatogonia. Isolated gonocytes (D,E,F) and spermatogonia (G,H,I) were fixed on slides and stained with germ cell markers. 95% of both gonocytes (1D) and spermatogonia (1G) expressed oct4, while less than 5% of gonocytes (1E) and spermatogonia (1H) expressed PLZF. UCHL1 was expressed in over 95% of gonocytes (1F) and spermatogonia (1I). The expression of PLZF, OCT3/4 and UCHL1 are consistent with previous reports for germ cells indicating our isolated cell populations contained 95% germ cells.

Gonocytes and spermatogonia were isolated from postnatal day one and day 7–9 Swiss male mice respectively. Whole testes were enzymatically dissociated and separated on a 2–4% continuous bovine serum albumin (BSA) gradient, as previously described [39]. Germ cell enrichment (>95%) was assessed by immunocytochemistry with germ/pluripotency/stem cell marker(s) PLZF (Promyelocytic Leukaemia Zinc Finger Protein), UCHL1 (ubiquitin carboxyl-terminal esterase L1), and OCT 3/4 (POU5F1, POU domain, class 5, transcription factor 1) (Fig. 1) [40], [41]. Briefly, cells were dried on multiwell slides (10,000 cells per well) and stored at −20°C until utilised. In preparation for probing with germ cell markers, the slides were thawed then rinsed in PBST (PBS, 0.1% Tween (Astral Scientific, Caringbah, NSW, Australia) before being blocked and the cells permeabilised with 1% BSA in PBSTx (PBS, 0.025% Triton X100) for 1 hour at room temperature. Cells were probed with the primary antibody overnight at 4°C (see Table S1 for antibody concentrations), then washed before the appropriate Alexa Fluor 594-conjugated fluorescent secondary antibody (Invitrogen) was applied for 1 hour at room temperature. The cells were washed and counterstained with 4′,6-diamidino-2-phenylindole (DAPI) (Invitrogen) before being mounted with anti-fade medium (Mowiol 4–88) and viewed on an Axio Imager A1 microscope (Carl Zeiss MicroImaging, Inc., Thornwood, NY. USA) equipped with epifluorescent optics and images captured with an Olympus DP70 microscope camera (Olympus America, Centre Valley PA. USA). Upon confirmation of target cell enrichment, populations of isolated spermatogonial cells were then either subjected to RNA or protein isolation as described below.

### Total RNA isolation and miRNA microarray analysis

RNA was extracted with phenol/chloroform as previously described [39] and incubated with 1%DNase (Promega), in accordance with the manufacturer's directions, to remove genomic DNA contamination. Total RNA (1.2 µg) was amplified and labelled for hybridization to the bead array matrix according to the manufacturer's instructions (Illumina, San Diego, CA, USA). All samples were analysed in biological triplicates. The array platform was the Illumina mouse miRNA microarray (using the version 1 revision 2 microRNA assay pool) covering 96% of the murine miRNA species described in the mirBase database release 12. The data was background subtracted and normalised against the mean of small nuclear RNA (snoRNA) 142 and snoRNA 234-1 using BeadStudio (version 3.0) software (Illumina). The miRNAs were listed according their relative abundance and the fold change between gonocytes and spermatogonia (Tables S2 and S3). The expression of each miRNA was clustered (Cluster3, Stanford University, Palo Alto, CA, USA [42]) and examined using heatmaps (Java Treeview, Stanford University, Palo Alto, CA, USA [43]) to visualise trends in miRNA expression in developing germ cells. Expression analysis was then undertaken with Significance Analysis of Microarrays (SAM) statistical software (academic version 2.23 http://www-stat.stanford.edu/_tibs/SAM/ [44]). SAM expression analysis was performed with a two-class unpaired Wilcoxon test on unlogged data using 500 permutations. Significant miRNA species had a q-value<4 (false discovery rate <4%). The array data described above has been deposited in the NCBI's Gene Expression Omnibus (GSE36566).

### Reverse transcription of miRNA and mRNA and qRT-PCR

For miRNA, 500 ng of total RNA was reverse transcribed with specific primers (Sigma) (1 µM), one control (U6) and 2 targets per reaction, and Superscript II (Invitrogen) according to the manufacturer's instructions. For mRNA, 3 µg of total RNA was reverse transcribed with oligo dT primers and reverse transcriptase (Promega) according to the manufacturer's instructions.

RT-PCRs were performed as previously described [39]. cDNA was amplified with GoTaq qPCR Master Mix (Promega), or 2×SYBR® GreenER™ qPCR SuperMix Universal (AB Gene, (Thermo Scientific, Rockford, IL, USA)) according to the manufacturer's protocol and amplification was conducted using an MJ Opticon 2 real time thermocycler (Bio-Rad, Hercules, CA, USA). The forward primers were used in conjunction with m13F to amplify the cDNA during a PCR reaction. Each PCR was performed on at least five separate cell isolations in the case of miRNA reactions and three cell isolations for mRNA reactions. qPCR data is shown as the average relative gene expression in spermatogonia compared to gonocytes using the 2e-ΔΔCT calculation as described previously [45]. In order to eliminate variation in the concentration of RNA used for the RT-reaction all PCR reactions were normalised to either small nuclear RNA6 (miRNA samples) or cyclophilin (mRNA samples).

To amplify the miRNA of interest specific primers were designed with the reverse primer consisting of the last 8 nucleotides in reverse complement of the miRNA sequence followed by the m13F sequence. This primer was used in the first strand synthesis during the RT reaction. The forward primer was the remainder of the miRNA sequence. In instances when the melting temperature of the forward primer was less than 50°C, the sequence was modified to include locked nucleic acids (GeneWorks, Hindmarsh, SA, Australia) to raise the melting temperature to at least 50°C. The forward primers were used in conjunction with m13F to amplify the cDNA during a PCR reaction. The blast function in the miRBase database was used to check primer specificity and providing the forward or reverse primer were specific they were considered valid for use. For the mRNA qRT-PCR reactions a list of the specific primers is included in the supplementary material (Table S4).

### miRNA target prediction and pathway identification

To obtain the most complete list of targets for the miRNA molecules of interest as well as limit the number of false positives we took advantage of the feature in the miRWalk database (http://www.ma.uni-heidelberg.de/apps/zmf/mirwalk/) that allows simultaneous searches of several databases [46]. We interrogated the following seven databases; miRanda, MiRDB, MiRWalk, PITA, RNA22, RNAhybrid and Targetscan. When four or more of these programs co-identified a specific transcript, then the target(s) were selected for our list of potential targets for closer examination. In addition, due to the limited ability of all algorithms to predict targets of miRNA complementary strands (*) these miRNA* targets were identified using miRWalk and MiRanda and only those targets predicted by both programs were examined more closely (Table S5). To gain a better understanding of the function of the up and down-regulated miRNAs they were co-analysed with Ingenuity Pathway Analysis version 8.8 (IPA, Ingenuity Systems, Redwood City, CA, USA) to identify the key effected pathways. Individual miRNA species analysis can be found in the supplementary materials (Figure S1).

### Protein extraction and Western blotting

Protein was extracted using 300 µl SDS lysis buffer (0.1% SDS, 375 mM Tris, pH 6.8, 1% sucrose), supplemented with a protease inhibitor cocktail (Protocease G-Biosciences St. Louis, MO, USA)) per 5 mg of cells or tissue. Protein concentration was estimated using a Pierce BCA Protein Assay Kit (Thermo Scientific, Rockford, IL, USA). SDS-PAGE and immunoblotting was conducted as previously described [39]. Membranes were “stripped” of primary and secondary antibodies using Western Re-Probe (G-Biosciences), according to the manufacturer's instructions and reprobed with anti-α-tubulin to confirm equal loading. Labelled proteins were detected with either ECL or ECL Plus Western blotting detection reagents according to the manufacturer's instructions (GE Healthcare, Buckinghamshire, UK). Densitometry was used to determine changes in protein expression in spermatogonia relative to gonocytes in two pooled samples by normalising against the loading control of α-tubulin. Using this system, higher expression in spermatogonia is indicated by a number greater than 1 while lower expression in the spermatogonia samples is indicated by a number less than 1.

### Immunohistochemistry

Tissue was fixed for 4 h in Bouin's fixative before being paraffin embedded and sectioned (4 µm). Slides were dewaxed and rehydrated before heat activated antigen retrieval in TE buffer (10 mM Tris, 1 mM EDTA) was performed. Slides were allowed to cool before being blocked (3% BSA in PBST) for 1 hour. Sections were probed with an appropriate primary antibody overnight at 4°C (Table S1), then washed before an Alexa Fluor 594-conjugated secondary antibody (Invitrogen) was applied for 1 hour at room temperature. The slides were washed and counterstained with DAPI before being mounted with anti-fade medium (Mowiol 4–88, Sigma) and viewed using epifluorescent microscopy as described above.

## Results

### Characterisation of enriched germ cell populations

At birth, mouse gonocytes are located in the centre of the seminiferous tubules. However, once differentiation into spermatogonia begins at postnatal day 2 these germ cells relocate at the basement membrane (Fig. 1A), where they become established in their niche - thus allowing them to commence the process of spermatogonial division and differentiation, see postnatal day 8 (Fig. 1B). In order to interrogate the changes in miRNA expression between gonocytes and spermatogonia, these cell populations were isolated and confirmed as germ cells. For this purpose, the unique cell populations were separated using an established BSA gradient sedimentation technique [39] and then examined for the expression of several germ cell markers. Analysis with qRT-PCR revealed a number of significant differences in the expression germ/pluripotency/stem cell markers, including *Oct3/4* (pluripotency marker) and *Ngn3* (early differentiating germ cell marker [47]) which were up-regulated in the spermatogonia cell population and, *Nanog* and *Plzf* (stem cell markers) which were down-regulated in the spermatogonia cell population (Fig. 1C). This serves to illustrate that a degree differentiation has occurred between day 1 and day 7–9 germ cells. At the protein level over 95% of the total cell population in both the gonocyte and spermatogonial cell enriched fractions were shown to express the pluripotency marker OCT3/4 (Fig. 1D, 1G), while only 3–6% of the cell population expressed the stem cell marker PLZF (Fig. 1E, 1H). Finally over 95% of the cell populations expressed the undifferentiated germ cell marker UCHL1 [41] (Fig. 1F, 1I) indicating a highly enriched germ cell population within these samples.

### Microarray and qPCR analysis of enriched germ cell populations

**Figure 2:**
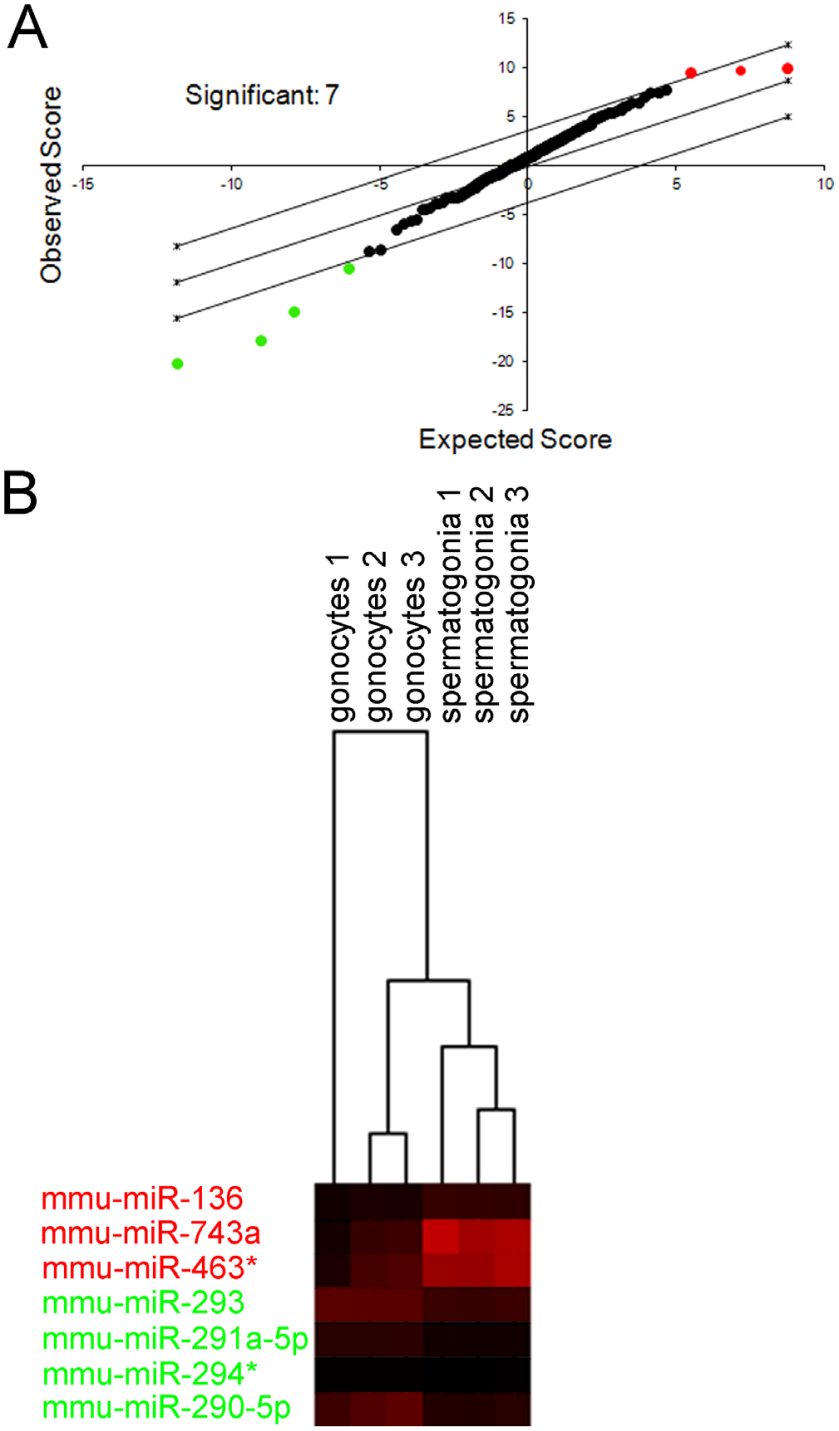
Data analysis of the miRNA microarray. Total RNA was isolated from gonocytes and spermatogonia (three biological replicates) and analysed using the Illumina mouse miRNA microarray (version 1 revision 2). A) Significance Analysis of Microarrays (SAM) statistical software output (academic version 2.23 http://www-stat.stanford.edu/_tibs/SAM/) [44] was used to identify significantly different miRNA molecules between gonocytes and spermatogonia. SAM expression analysis was performed with a two-class unpaired Wilcoxon test on unlogged data using 500 permutations. Significant miRNA species had a q-value<4 (false discovery rate <4%). Seven miRNA molecules were identified by SAM; 3 were up-regulated in spermatogonia (red) while 4 were down-regulated (green). B) The expression of the significant miRNAs in the individual replicates used for the array was examined using a heat map (Java Treeview) red staining indicating higher expression. miRs 136, 743a and 463* were expressed at consistently higher levels in the spermatogonia samples and the expression of miRs 293, 291a-5p, 294* and 290-5p were expressed at consistently lower levels in spermatogonia.

On verification of a highly enriched population of germ cells, we characterised the differences in miRNA expression between gonocytes and spermatogonia. For this purpose, total RNA was extracted from gonocyte and spermatogonial cell populations (n = 3 biological samples) and hybridised to a mouse Illumina bead microarray as outlined in the materials and methods. The array data was normalised and the miRNA molecules ranked according to their expression (Table S2), and fold change, from gonocytes to spermatogonia (Table S3). However, to discover miRNA with significantly different expression profiles between these cell populations, SAM statistical software was utilised. This program identified three significantly up-regulated (miR-136, 743a and 463* (q-value% = 3.811) and four significantly down-regulated miRNA species (MiR-293, 291a-5p, 290-5p and 294* q-value% = 0) between gonocytes and spermatogonia (Fig. 2A). The expression of these miRNA molecules was examined in the individual biological samples using a heat map (Fig. 2B). Notwithstanding some variation between the biological samples, this approach confirmed that these miRNA molecules were differentially expressed between gonocytes and spermatogonia.

**Figure 3:**
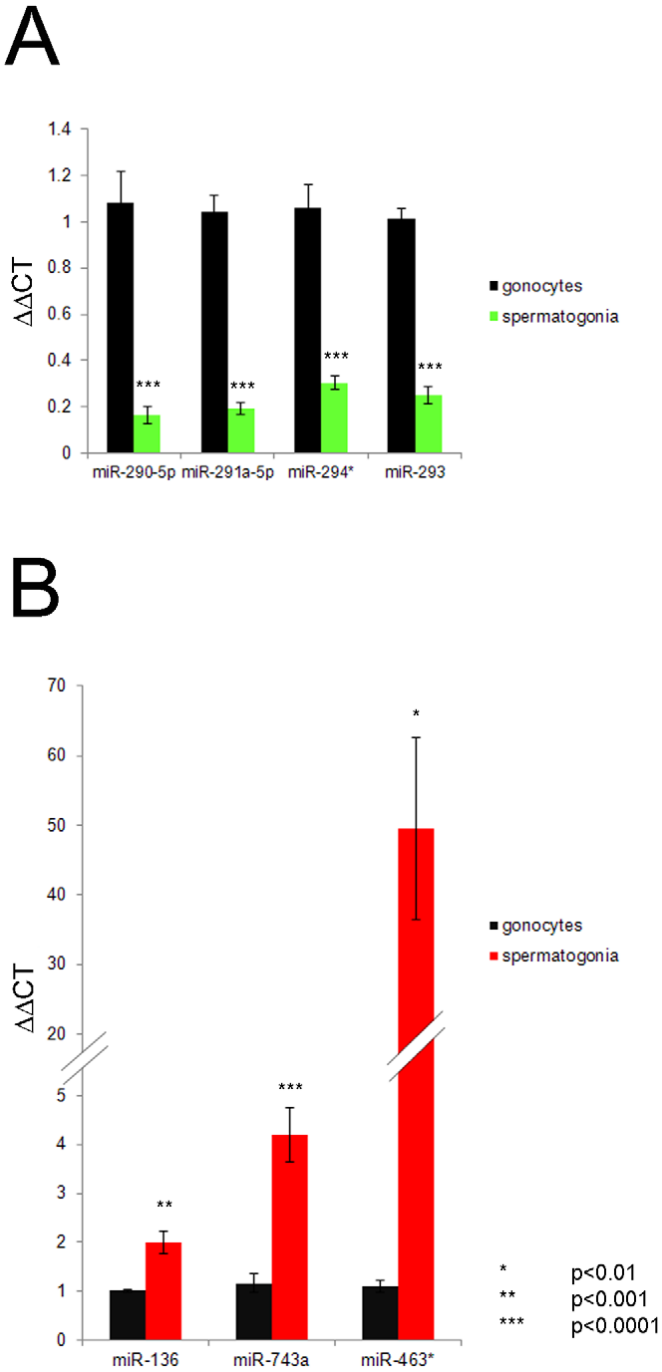
Confirmation of miRNA microarray analysis using qPCR. Total RNA from isolated germ cell populations (five biological replicates) was reverse transcribed with specific primers and specific miRNA molecules were amplified by QPCR. The resultant data was analysed using the 2e-ΔΔT calculation to determine the expression in spermatogonia relative to gonocytes. A) All the down-regulated miRNA (miR-293, 291a-5p, 294* and 290-5p (green)) identified by the SAM analysis in Figure 2 were found to be expressed in spermatogonia at approximately 20% of the level they were in gonocytes (p<0.0001). B) All the down-regulated miRNA (miR-136, 743a and 463* (red)) identified by SAM were found to be expressed at significantly higher levels in spermatogonia than in gonocytes. miR-136 was up-regulated 2 fold (p<0.001), miR743a was up-regulated 4 fold (p<0.0001) while miR-463* was up-regulated 50 fold (p<0.01).

The expression of the seven miRNA molecules identified by SAM was further validated through use of qPCR across five biological samples. As anticipated, this analysis confirmed that each of the seven target miRNAs were indeed differentially expressed between the two cell types. The down-regulated miRNA molecules, miR-293, 291a-5p, 290-5p and 294* were each expressed at levels that were approximately five fold lower in spermatogonia compared to gonocytes (p<0.0001) (Fig. 3A). Although the up-regulated targets were also confirmed as significant, the relative fold change in expression of these individual miRNA species varied dramatically. In this regard, miR-136 was expressed in spermatogonia at approximately twice the level it was detected in gonocytes (p<0.001), while miR-743a exhibited a fourfold change (p<0.0001) and miR-463* was elevated to approximately 50 fold the levels observed in gonocytes (p<0.01) (Figure 3B). In the case of miR-463* absolute expression was low resulting in greater variation when expressed in relative terms, although the differences were significant.

### Identification and characterisation of targets of significant miRNA

**Figure 4:**
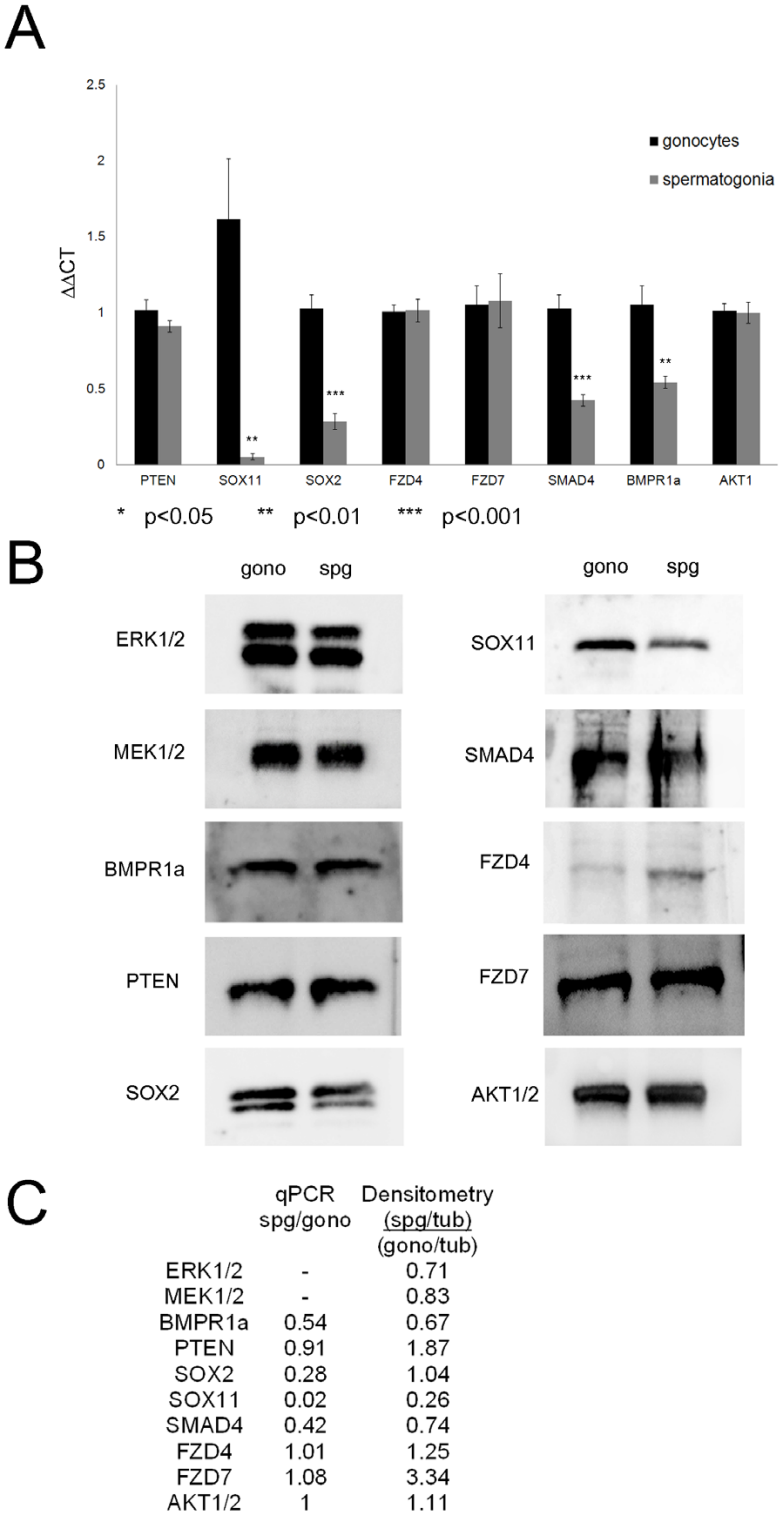
Expression of predicted targets of up and down-regulated miRNA molecules. A) Total RNA from isolated gonocytes and spermatogonia (three biological replicates) was reverse transcribed before gene specific amplification. The resultant data was analysed using the 2e-ΔΔT calculation to determine mRNA expression in spermatogonia relative to gonocyte expression. Gene expression of *Sox11* (p<0.01) and *Sox2* (p<0.001), *BMPR1a* (p<0.01) *SMAD4* (P<0.001) was reduced in the spermatogonia. The expression of the other genes examined: *PTEN*, *FZD4*, *FZD7* and *AKT1* did not change between gonocytes and spermatogonia. B) SDS protein extractions of isolated gonocyte and spermatogonia cells were analysed by SDS-PAGE and immunoblotting [39]. C) The mRNA and protein expression of genes of interest expressed relative to gonocyte expression. mRNA expression was determined via qPCR (values from A). Densitometry was used to quantify the protein expression between gonocytes and spermatogonia (n = 2). A ratio of the protein of interest to the α TUBULIN control was used to normalise loading. The ratio of spermatogonia to gonocyte demonstrates the change in protein expression. A value greater than 1 indicates higher expression in the spermatogonia while a value less than 1 indicates higher expression in gonocytes.

Having identified significant changes in the miRNA expression profile between gonocytes and spermatogonia, we sought to determine the functional significance of each of the seven miRNA species. As described in materials and methods, targets predicted by multiple software programs were selected for further investigation. Lists of targets for each individual miRNA molecule can be found in the supplementary material (Table S5). Among the large number of putative targets identified by this approach, a number were selected for closer examination on the basis of established roles in spermatogenesis, carcinogenesis, key cell signalling pathways or stem cell maintenance. These included *Pten (Phosphate and Tensin Homolog)*, *Sox2 (Sex Determining Region Y Box 2)*, *Sox11 (Sex Determining Region Y Box 11), Fzd4 (Frizzled Receptor 4)* and *Fzd7* (*Frizzled Receptor 7*), *Bmpr1a (Bone Morphogenetic Protein Receptor Type 1a)*, *Smad4 (Sterile Alpha Motif Domain Containing 4)*, *Mek (MAP kinase kinase)*, *Erk (MAP kinase)* and *Akt*. The expression of the majority of these candidate targets mRNA's was examined by qPCR (Fig. 4A) in three biological samples. We determined that the expression of *Fzd4*, *Fzd7*, *Pten*, and *Akt* mRNA, with the exception of *Fzd7*, all targets of significantly down-regulated miRNAs, did not change appreciably during the differentiation of gonocytes into spermatogonia. The mRNA expression of *Bmpr1a* (p = 0.0012), *Sox*2 (p<0.0001), *Sox11* (p = 0.0012) and *Smad4* (p<0.0001), with the exception of *Sox2*, all targets of significantly up up-regulated miRNAs, were significantly down-regulated in spermatogonia, suggesting that they may be the target of miRNA mediated transcript degradation. However, given that regulation of gene expression by miRNA can also affect mRNA translation, we also examined the protein expression levels targets in gonocytes and spermatogonia via immunoblotting (Fig. 4B). Using densitometric analysis in duplicate to normalise for protein loading (Fig. 4C), it was determined that the SOX11 protein was expressed at much lower levels in spermatogonia (0.26 times) than in gonocytes. This matches the expression seen in the mRNA analysis. In accordance with the quantitation of mRNA, but to a lesser degree, the protein expression of BMPR1a (0.67 times) and SMAD4 (0.74 times) was lower in spermatogonia than gonocytes. SOX2 protein was expressed at equivalent levels between gonocytes and spermatogonia, in contrast to the declining transcript expression in spermatogonia in the qPCR analysis (Fig. 4A). The disparity between the PTEN transcript and protein expression was attributed to decreased translational repression by miRNAs occurring in spermatogonia, causing higher expression levels to occur (1.87 times). Similarly, post-transcriptional control may contribute to the observed elevation in FZD7 (3.34 times) and FZD4 (1.25 times) protein expression in spermatogonia compared to gonocytes. The protein expression of AKT was similar between gonocytes and spermatogonia, and corresponded to the observed transcript levels. Two further downstream targets were examined and protein expression of ERK1/2 (0.71 times) and MEK1/2 (0.83 times) were also found to be slightly lower in spermatogonia than gonocytes.

**Figure 5:**
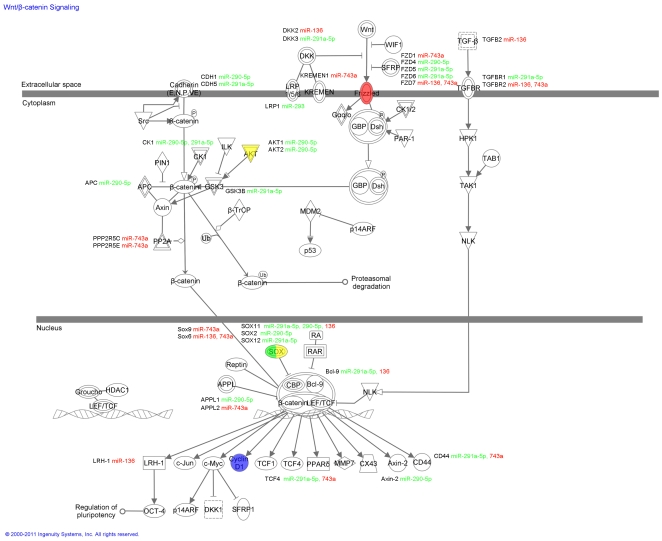
Wnt/β catenin signalling pathway. The Wnt/β catenin signalling pathway was chosen from the pathways identified in the IPA analysis (Figure S2) for closer examination. The signalling pathway was outlined in IPA and the genes targeted by miRNA molecules are identified on the pathway alongside their targeting miRNA molecules (red: up-regulated in spermatogonia, green: down-regulated in spermatogonia). Targets examined for gene and protein expression levels are colour coded according to their expression in gonocytes and spermatogonia, up-regulated molecules red, unchanged molecules yellow and down-regulated molecules green. In this pathway the expression of AKT, fzd4 and fzd7 was unchanged. Sox2 and sox11 expression were down-regulated at both the gene and protein level. The target protein Cyclin D1 is identified by blue highlighting.

**Figure 6:**
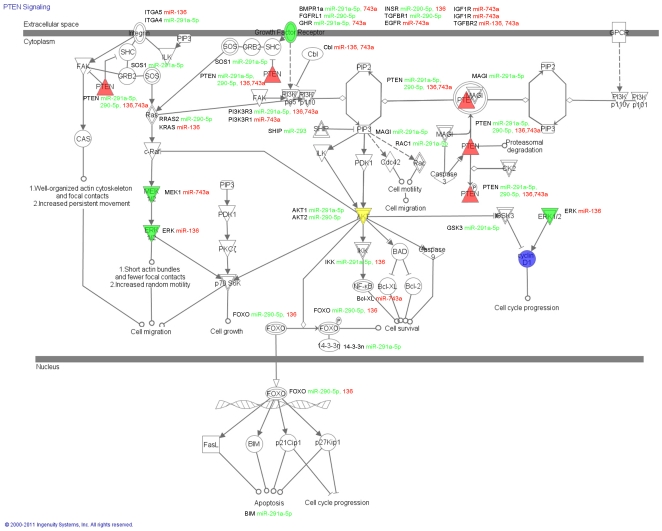
The PTEN signalling pathway. The PTEN signalling pathway was chosen from the pathways identified in the IPA analysis (Figure S2) for closer examination. The signalling pathway was outlined in IPA and the genes targeted by miRNA molecules are identified on the pathway alongside their targeting miRNA molecules (red unregulated in spermatogonia, green down-regulated in spermatogonia). Targets examined for gene and protein expression levels are colour coded according to their expression in gonocytes and spermatogonia, up-regulated molecules red, unchanged molecules yellow and down-regulated molecules green. In the PTEN pathway the expression of PTEN, AKT, ERK and MEK remained unchanged between gonocytes and spermatogonia while the expression of the growth receptor BMPR1a was significantly reduced in spermatongia when compared to gonocytes. The target protein Cyclin D1 is identified by blue highlighting.

After determining that a number of key signalling molecules change significantly during gonocyte differentiation, we sought to determine which pathways had the potential to be most influenced by the seven miRNA molecules identified previously. This analysis was conducted by combining all of the potential targets of the up and down-regulated miRNAs (as identified by miRWalk) into two lists, which were used to interrogate the Ingenuity Pathway Analysis software to identify the pathways containing the most targets. The list of each miRNAs potential targets was also examined separately (Fig. S1). Within the top 15 pathways targeted by the miRNA which are down-regulated in spermatogonia (Fig. S2A) were several cancer metabolism pathways and immune regulatory pathways, and included both the PTEN and Wnt/β-catenin signalling pathways. Several similar pathways were also found in the top 15 molecular pathways targeted by up-regulated miRNAs (Fig. S2B), including the molecular mechanisms of cancer and the PTEN signalling pathway. Other key pathways include immune regulation, metabolism, and retinoic acid receptor activation. Given that several of the identified protein targets are involved in the Wnt/β-catenin signalling pathway (Fig. 5) and/or the PTEN signalling pathway (Fig. 6) we elected to focus on these pathways and their potential to be regulated by our target miRNAs.

In Figure 5 the Wnt/β-catenin pathway is presented in full, with the members targeted by miRNAs indicated in the adjacent text with down-regulated miRNA molecules (in spermatogonia) in green and those up-regulated in red. Some members of the Wnt/β-catenin signalling pathway which are differentially expressed between gonocytes and spermatogonia such as the SOX proteins (see Fig. 4) are highlighted within this pathway, with down-regulated members green, up-regulated in red and those whose expression remained unchanged in yellow.

The possibility of miRNA regulation at multiple distinct points in the signalling cascade of the PTEN signalling pathway is high as is demonstrated in Figure 6, where predicted miRNA target(s) are shown. Several members of the PTEN pathway were differentially expressed between gonocytes and spermatogonia (see Fig. 4) and these are highlighted within the pathway, as described above for the Wnt/β-catenin signalling pathway.

**Figure 7:**
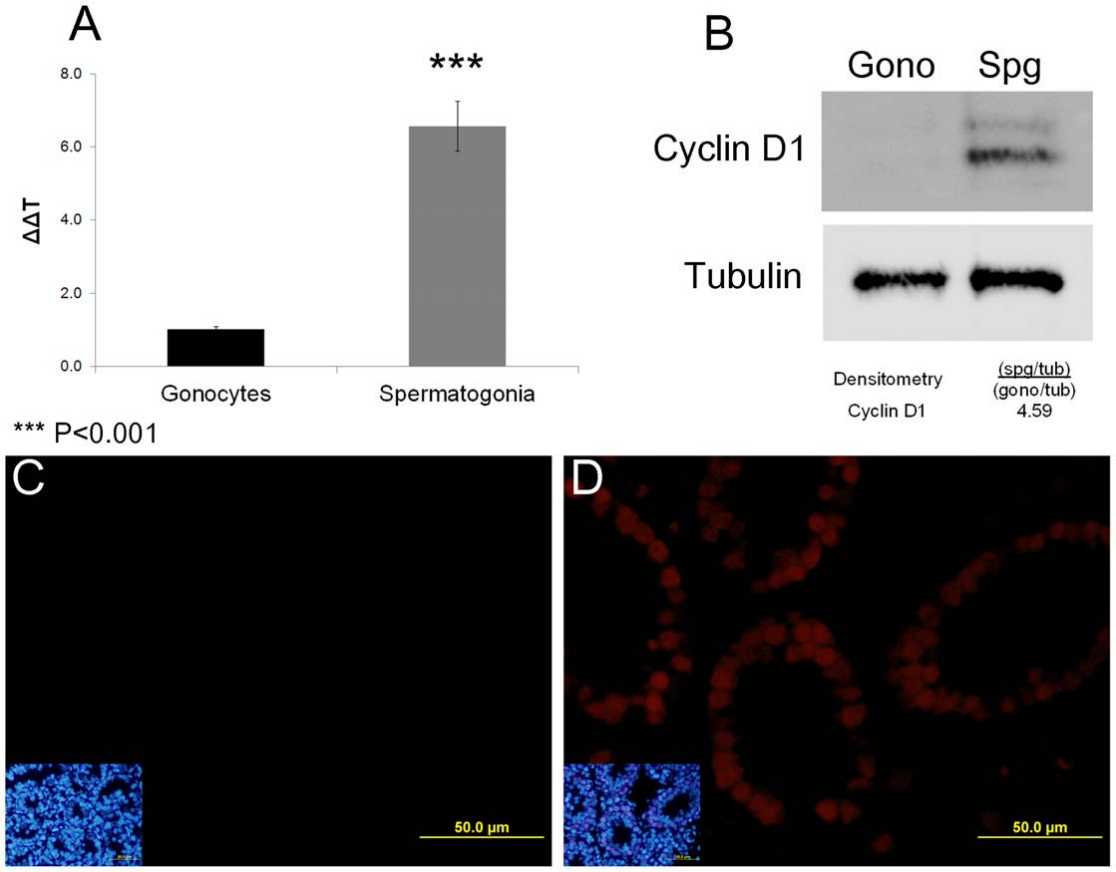
The expression of Cyclin D1 in gonocytes and spermatogonia. A) Total RNA from isolated gonocytes and spermatogonia (three biological replicates) was reverse transcribed before gene specific amplification. The resultant data was analysed using the 2e-ΔΔT calculation to determine mRNA expression in spermatogonia relative to gonocyte expression. Cyclin D1 miRNA expression in spermatogonia was found to be 6 times higher than in gonocytes (p<0.0001). B) SDS protein extractions of isolated gonocyte and spermatogonia cells were analysed by SDS-PAGE and immunoblotting [39]. Densitometry analysis of immunoblots (n = 2) indicates that the expression of Cyclin D1 was four times higher in spermatogonia compared to gonocytes. Immunohistochemistry of Cyclin D1 (red) on day 2 (Figure 7C) and day 8 (Figure 7D) indicates that Cyclin D1 was not expressed in day 2 testis but was expressed in the spermatogonia cells of day 8 testis.

In terms of the Wnt/β-catenin pathway (Fig. 5) there are a number of potential candidates that may be suppressed and/or activated by our target miRNAs. A key downstream target of this pathway that may be indirectly regulated by the differentially expressed miRNA identified in this study is Cyclin D1. Indeed, the positive regulator of Cyclin D1 within the PTEN pathway (Fig. 6), ERK1/2, was targeted by miR-136 (up-regulated molecule) while the negative regulator, GSK3, was targeted by miR-291a-5p (down-regulated molecule). On the basis of these findings we examined the expression of Cyclin D1 mRNA and protein in gonocytes and spermatogonia using qPCR, immunoblotting and immunohistochemistry (Fig. 7). These analyses revealed that Cyclin D1 was expressed in spermatogonia at much higher levels than in gonocytes. Cyclin D1 transcript expression was approximately six fold higher in spermatogonia than that of gonocytes (Fig. 7A). Consistent with such findings, Cyclin D1 protein was undetectable in gonocytes (Fig. 7B,C). It was however clearly detected in cell lysates prepared from spermatogonia and was localised to the nucleus of these cells. These data support the notion that Cyclin D1 is involved in the differentiation of gonocytes and/or the proliferation of spermatogonia and afford proof of principle that differential miRNA expression may be a key regulator of these important processes.

## Discussion

In our study of differentially expressed miRNA all the significantly down-regulated miRNA molecules i.e. miR-293, 291a-5p, 290-5p and 294* belong to the miR-290-295 cluster and have previously been found to be highly enriched in the germ cell population of day 6 testis when compared to the somatic cell population [48]. Additionally, the miR-290-295 cluster is highly expressed in mouse embryonic stem cells [49], with a role in promoting cell proliferation and maintaining pluripotency. This cluster is a target of pluripotency factors, and in turn targets the pluripotency factors OCT4, SOX2 and NANOG [49]–[51]. The artificial expression of members of the miR-290 cluster (291a-3p, 294 and 295) restores the proliferation potential of ES cells lacking members of the miRNA processing machinery. These ES cells have a tendency to stall in the G1 stage of the cell cycle [52]. More recently the miR-290 cluster has been found to have a pro-survival role by directly targeting pro-apoptosis genes, Caspase2 and Ei24 for mRNA degradation [49]. The expression of the miR-290 cluster is high in multipotent adult germ cells, which are established upon culture of spermatogonial stem cells under ES cell conditions. We propose that the elevated expression of the miR-290 cluster maintains the expression of the pluripotency factor OCT4, and promotes the expression of early germ cell specific proteins in these cells for an extended period when compared to ES cells stimulated to differentiate [50], [53]. Additionally, knockout of the miR-290 cluster, besides inducing a partially penetrant embryonic lethality, leads to the defective migration of primordial germ cells to the genital ridges during development, resulting in reduced germ cell numbers in the nascent gonads. Male germ cells recovered their numbers by mitotic proliferation while female germ cells were unable to do this causing infertility in the surviving female knockouts [51]. The reduction in the expression of the members of the miR-290 cluster in our spermatogonial analysis, we believe, indicates that these cells have initiated the process of differentiation i.e. spermatogenesis.

In contrast, little is known about the three miRNAs (miR-136, miR-743a and miR-463*) up-regulated in spermatogonia and their role in germ cell development remains unclear. The miR-136 has been found to be highly expressed in placental tissue as well as overexpressed in lung cancer, however knockdown has no effect on tumour growth, and its function has still not been elucidated [54], [55]. Conversely miR-743a has been identified to play a role in the oxidative stress response in mitochondria [56]. The above miRNAs are only slightly enriched in germ cells (miR-743a and 463*) or preferentially expressed in the somatic tissue (miR-136) of day 6 testis [48] indicating that these miRNA molecules may play a role in the differentiation process and not in maintaining pluripotency.

One pathway identified in spermatogonia by our bioinformatic analysis (Fig. S2) as containing a significant number of differentially expressed miRNA targets was the Wnt signalling pathway (Fig. 5). Wnt signalling has been shown to play a role in both stem cell renewal and differentiation in response to differing signals from surrounding niche cells [57]. Wnt signalling also participates in the maintenance of the undifferentiated spermatogonial cell population and promoting their proliferation [57]. Treatment of isolated early male germ cells with Wnt3a and Wnt10b promotes morphological changes and cell migration, indicating Wnt signalling could have unique roles in addition to directing spermatogonial stem cell renewal [57]. Knockout of naked cuticle 1 (Nkd1), an antagonist of the canonical Wnt signalling pathway (controlling novel gene expression), caused sub fertility in male mice. The elongating spermatid population is reduced in these mice, indicating a problem in the later stages of spermiogenesis [58]. Furthermore, elements of the non-canonical Wnt signalling pathway i.e. Dishevelled1 (Dvl1) (which influences cell morphology and migration), are thought to control the morphological change(s) of spermatids that eventually permit their differentiation into mature spermatozoa [59]. In addition to their essential role in germ cell development, Wnt signalling also directs the maturation of Sertoli cells and their ability to support the process of spermatogenesis [60]. Wnt signalling is involved in the maintenance of “stemness” in multipotent and pluripotent stem cells including neural, mammary and embryonic stem cells [61]. As components of the Wnt signalling pathway such as FZD7 and FZD4 are disrupted in many cancer types including colon, renal, brain and breast tumours [62]–[64], it is believed they promote the ability of the tumour to proliferate and invade the surrounding tissue. The multiple potential miRNA targets identified in the Wnt signalling pathway in our analysis indicates regulation by miRNA molecules is likely to play a significant role in modifying the Wnt signalling pathway and in turn germ cell development. By analogy it is possible that dysregulation of the miRNAs may contribute to germ cell tumour development.

Within the Wnt signalling pathway (Fig. 5) the expression of Sox2 and Sox11 was examined and were found to be decreased in spermatogonial cells when compared to gonocytes (protein and/or mRNA) (Fig. 4). As described previously, Sox2 is involved in the maintenance of pluripotency [49]–[51]. While the role of Sox11 is not as well defined as that of Sox2, particularly in postnatal life, it has been implicated in neuron development and differentiation in the embryo [65]. Sox11 has also been found to be expressed in multipotent stromal stem cells, and Sox11 knockdown leads to reduced proliferation and promotes differentiation [66]. In contrast, knockdown of Sox11 in mantle cell lymphoma cell lines actually increases cell proliferation and promotes tumorigenesis when injected into nude mice [67]. Therefore, while it is clear that Sox11 is involved in the regulation of the cell cycle, its ability to promote or inhibit cellular proliferation may in fact be influenced by the specific cellular environment in which it is expressed. In the case of spermatogonia, the reduction in Sox11 expression may increase cell proliferation by up regulating the expression of the downstream target Cyclin D1 (Fig. 5 and 7). Interestingly, this same protein also appears as a downstream target of the PTEN signalling cascade, suggesting convergence or cross-talk between these pathways may be important in driving the differentiation of gonocytes.

The PTEN tumour suppressor pathway was also predicted to contain members which were potential targets of differentially expressed miRNA (Fig. S2 and 6). One of the possible outcomes from the PTEN signalling pathway is the modification of the cell cycle via the post translational regulation of Cyclin D1. Cyclin D1 is known to promote cell cycle progression from G_1_ to S phase [68], and its expression has previously been shown to occur in spermatogonia at post natal day 4 when the cell cycle has resumed [69]. After post natal day 4 in the mouse testis, we propose that post-translational control of the activity of Cyclin D1 could be required to fine tune the cell cycle. The seven significantly different miRNA molecules between gonocytes and spermatogonia identified in this study have the potential to influence the activity of Cyclin D1. One down-regulated miRNA molecule (miR-291a-5p) targets the negative regulator of Cyclin D1 (GSK3) allowing its expression and therefore limiting the effect of Cyclin D1. At the same time the positive regulator of Cyclin D1, ERK1/2, is also theoretically targeted by an up-regulated miRNA molecule (miR-136), which may result in a reduction of expression to limit the effect of Cyclin D1 on the cell cycle.

Cyclin D1 has previously been shown to be an indirect target of miRNA via a different pathway. Using epithelial cells transformed by c-Myc, Feng et al [70] demonstrated miR-378 was a direct target of c-Myc. miR-378 in turn cooperates with either RAS or HER2 to target the cell cycle repressor TOB2 which directly controls the expression of Cyclin D1 [70]. As Cyclin D1 is frequently over expressed in testicular germ cell tumours which have become resistant to cisplatin therapies, we concur with the hypothesis that the high levels of Cyclin D1 cause an accelerated cell cycle transition and limit the cells sensitivity to the chemotherapy agent [68], [71].

The KIT and EBBR2 receptors are frequently up regulated in testicular germ cell tumours. Both of these receptors activate the KRAS signalling pathway, which in turn is also commonly up-regulated in these tumours. KRAS activates both the MAP kinase pathway as well as the PI3K/AKT pathway to control cell proliferation and survival [72]. The PI3K/AKT is a key signalling pathway promoting cell proliferation, survival and migration which is subject to modulation by several negative regulators such as PTEN and PI3kip1 [73], [74]. Importantly, the ablation of PTEN is associated with the transformation of CIS cells, the precursor lesion for seminoma and nonseminoma into invasive cancer [73]. PTEN loss allows these cells to survive and proliferate without the support of the Sertoli cells, a key step in the neoplastic development of CIS [73]. In addition, the reduced expression of PI3kip1 in testicular germ cell tumours is associated with a high disease relapse rate [74]. PI3K and AKT are activated by GDNF and have been shown to be key promoters of self- renewal and survival in spermatogonial stem cells [75] as well as promoting the proliferation and survival of meiotic germ cells. However, despite PTEN being expressed in all stages of male germ cells and very highly expressed in spermatogonia, the PTEN knockout mouse has normal spermatogenesis, are fertile and have no testicular tumours, indicating that PTEN may have a functional redundancy in mice [76]. Knockdown of ETV5 and POU3F1 (oct6), both transcription factors activated by GDNF, caused apoptosis in spermatogonial stem cells in part due to the overexpression of PI3kip1, further indicating the importance of PI3K/AKT signalling and its regulation in normal spermatogenesis [77]

In this study we identified seven miRNA molecules differentially expressed between postnatal gonocytes and spermatogonia. The miRNA molecules down-regulated (miR-293, 291a-5p, 290-5p and 294*) are located in a miRNA cluster previously found to be involved in the maintenance of pluripotency within stem cells. In contrast, the up-regulated miRNA molecules in spermatogonia (miR-136, MiR-743a and miR-463*) have mostly unknown functions. However, the abundance of predicted targets in the Wnt/β-catenin signalling pathway and the PTEN signalling which have previously been found to be important for male fertility indicates their possible role in maintaining germ cell differentiation and or pluripotency.

## Supporting Information

Figure S1 Top pathways identified by IPA with potential to be affected by significant miRNA. Individual lists of targets as shown in supplementary table S5 of significant miRNA species were analysed by Ingenuity Pathway Analysis (IPA) version 8.8 to identify the effected pathways. IPA analysis output graphs show the measure of significance (right tailed Fishers exact test (P value logged) in the histogram bars while the proportion of targeted proteins over total proteins in the pathway is shown as a line. A) The top 15 pathways identified as containing a high number of targets miR-136. B) The top 15 pathways identified as containing a high number of targets of miR-463*. C) The top 15 pathways identified as containing a high number of targets of miR-743a. D) The top 15 pathways identified as containing a high number of targets of miR-290-5p. E) The top 15 pathways identified as containing a high number of targets of miR-293. F) The top 15 pathways identified as containing a high number of targets of miR-294*. G) The top 15 pathways identified as containing a high number of targets of miR-291a-5p.
(TIF)

Figure S2 Top pathways identified by IPA with potential to be affected by significant miRNA. Targets of significant miRNA molecules were predicted as described in the text (or in materials and methods) Combined targets of up or down-regulated miRNA species were then analysed by Ingenuity Pathway Analysis (IPA) version 8.8 to identify the effected pathways. IPA analysis output graphs show the measure of significance (right tailed Fishers exact test (P value logged) in the histogram bars while the proportion of targeted proteins over total proteins in the pathway is shown as a line. A) The top 15 pathways identified as containing a high number of targets of miRNA down-regulated in spermatogonia. B) The top 15 pathways identified as containing a high number of targets of miRNA up-regulated in spermatogonia.
(TIF)

Table S1 Antibodies in use and conditions for immunoblots, immunocytochemistry and immunohistochemistry.
(DOC)

Table S2 miRNA molecules in gonocytes and spermatogonia ranked according to their expression levels. Total RNA from highly enriched gonocytes and spermatogonia was labelled and hybridized to the Illumina mouse miRNA microarray (version 1 revision 2). The data was backgrounds subtracted and normalised against the mean of (small nuclear RNA) snoRNA 142 and snoRNA234-1. The miRNA was then ranked according to its abundance. A) miRNA species ranked according to their expression in gonocytes. With the most highly expressed miRNA species appearing first. B) miRNA species ranked according to their expression in spermatogonia. With the most highly expressed miRNA species appearing first.
(XLS)

Table S3 miRNA molecules ranked according to their fold change between gonocytes and spermatogonia. Total RNA from highly enriched gonocytes and spermatogonia was labelled and hybridized to the Illumina mouse miRNA microarray (version 1 revision 2). The data was backgrounds subtracted and normalised against the mean of (small nuclear RNA) snoRNA 142 and snoRNA234-1. The miRNA was then ranked according to its fold change between gonocytes and spermatogonia (gonocytes/spermatogonia).
(XLS)

Table S4 qPCR primer sequences and annealing temperatures. Forward and reverse primer sequences (Sigma) used to selectively amplify the genes of interest with their associated annealing temperatures. For the miRNA sequences capital letters indicate the location of locked nucleic acids used to raise the melting temperature of the primer (GeneWorks, Hindmarsh, SA, Australia).
(DOC)

Table S5 Complete list of predicted targets for miRNA molecules differentially expressed between gonocytes and spermatogonia. Targets for the miRNA molecules of interest were predicted by the feature in the miRWalk database (http://www.ma.uni-heidelberg.de/apps/zmf/mirwalk/) that allowed simultaneous searches of many databases. The databases included miRanda, MiRDB, MiRWalk, PITA, RNA22, RNAhybrid and Targetscan. The targets (rows) identified by a program (columns) are coloured green while the targets not identified by a program are red. Targets identified four or more of these programs were added to our list of potential targets that would be examined more closely. Genes are ranked according to the number of software programs predicting them as targets. However due to the limited algorithms able to predict targets of miRNA complementary strands (*) miRNA* targets were identified using miRWalk and MiRanda and those targets predicted by both these programs were examined more closely not colour coded. A) Targets for mmu-miR-136. B) Targets for mmu-mir-463*. C) Targets for mmu-miR-743a. D) Targets for mmu-miR-291a-5p. E) Targets for mmu-miR-293. F) Targets for mmu-miR-294*. G) Targets for mmu-miR-290-5p.
(XLS)
