# Supplementary figures and images for: A Unique Combination of Male Germ Cell miRNAs Coordinates Gonocyte Differentiation
Source: PLoS One. 2012 Apr 20;7(4):e35553. doi: 10.1371/journal.pone.0035553 (PMC3334999; doi:10.1371/journal.pone.0035553)

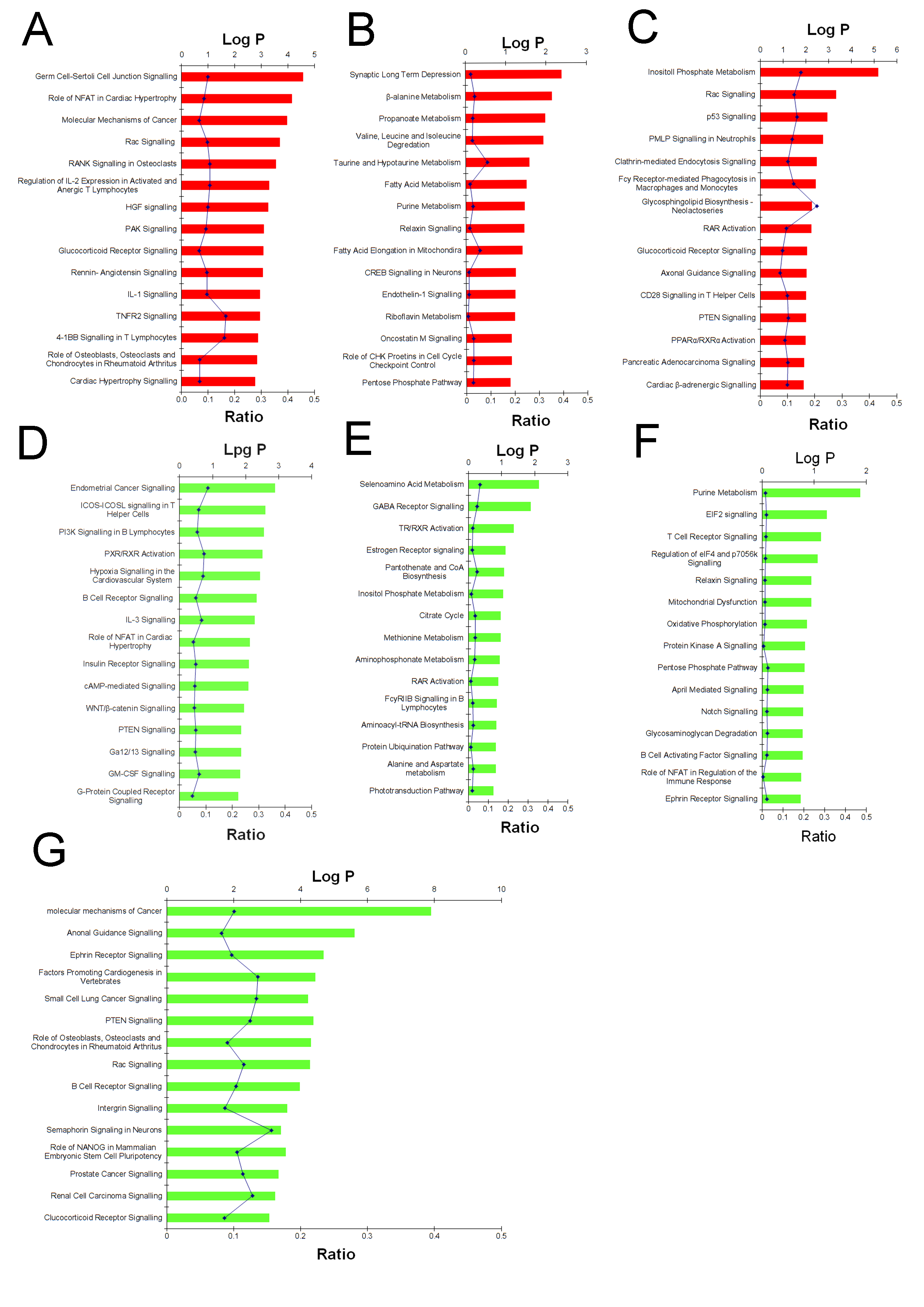

Supplement: Figure S1 — Top pathways identified by IPA with potential to be affected by significant miRNA. Individual lists of targets as shown in supplementary table S5 of significant miRNA species were analysed by Ingenuity Pathway Analysis (IPA) version 8.8 to identify the effected pathways. IPA analysis output graphs show the measure of significance (right tailed Fishers exact test (P value logged) in the histogram bars while the proportion of targeted proteins over total proteins in the pathway is shown as a line. A) The top 15 pathways identified as containing a high number of targets miR-136. B) The top 15 pathways identified as containing a high number of targets of miR-463*. C) The top 15 pathways identified as containing a high number of targets of miR-743a. D) The top 15 pathways identified as containing a high number of targets of miR-290-5p. E) The top 15 pathways identified as containing a high number of targets of miR-293. F) The top 15 pathways identified as containing a high number of targets of miR-294*. G) The top 15 pathways identified as containing a high number of targets of miR-291a-5p. (TIF) [file pone.0035553.s001.tif]

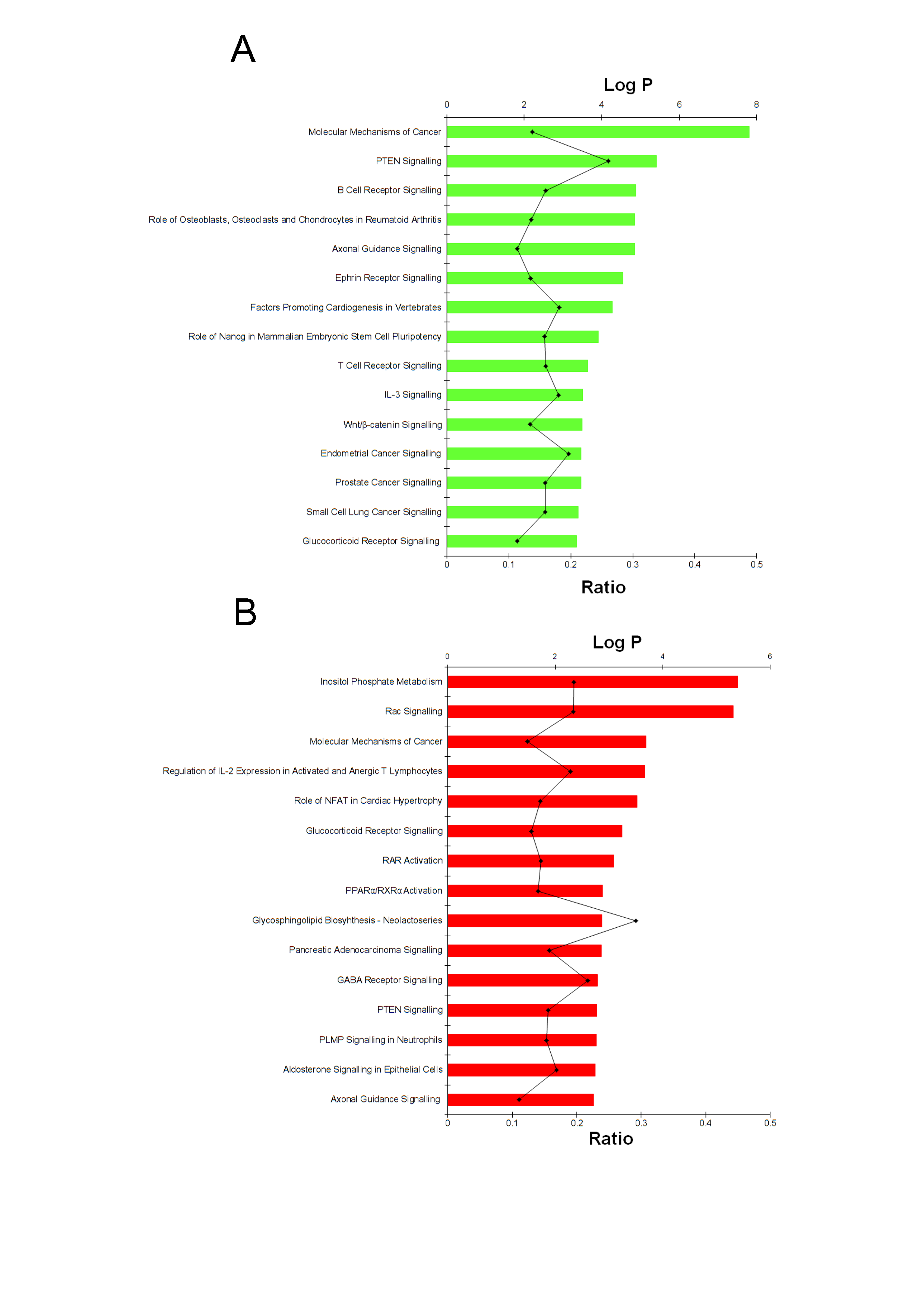

Supplement: Figure S2 — Top pathways identified by IPA with potential to be affected by significant miRNA. Targets of significant miRNA molecules were predicted as described in the text (or in materials and methods) Combined targets of up or down-regulated miRNA species were then analysed by Ingenuity Pathway Analysis (IPA) version 8.8 to identify the effected pathways. IPA analysis output graphs show the measure of significance (right tailed Fishers exact test (P value logged) in the histogram bars while the proportion of targeted proteins over total proteins in the pathway is shown as a line. A) The top 15 pathways identified as containing a high number of targets of miRNA down-regulated in spermatogonia. B) The top 15 pathways identified as containing a high number of targets of miRNA up-regulated in spermatogonia. (TIF) [file pone.0035553.s002.tif]
