# Supplementary material for: A Unique Combination of Male Germ Cell miRNAs Coordinates Gonocyte Differentiation
Source: PLoS One. 2012 Apr 20;7(4):e35553. doi: 10.1371/journal.pone.0035553 (PMC3334999; doi:10.1371/journal.pone.0035553)
Supplement: Table S1 — Antibodies in use and conditions for immunoblots, immunocytochemistry and immunohistochemistry. (DOC) [file pone.0035553.s003.doc]

Table S1: Antibodies in use and conditions for immunoblots, immunocytochemistry and immunohistochemistry.

| Protein | Catalogue # and company | IB block and dilution factor | ICC block and dilution factor | ICH antigen retrieval , block and dilution factor |
| --- | --- | --- | --- | --- |
| OCT3/4 | Ab27985, Abcam | NA | 1% BSA, 1:20 | NA |
| UCHL1 | Ab10404; Abcam | NA | 1% BSA, 1:20 | NA |
| PLZF | Op128; Calbiochem | NA | 1%BSA, 1:10 | NA |
| ERK1/2 | M5670; Sigma | 5% milk; 1:1000 | NA | NA |
| MEK1/2 | M5795; Sigma | 5% milk; 1:1000 | NA | NA |
| BMPR1a | Ap2004b Biocore | 5% milk;, 1:500 | NA | NA |
| PTEN | Sc7947; Santa Cruz | 5% milk; 1:500 | NA | NA |
| SOX2 | Ab42635; Abcam | 5% milk; 1:2000 | NA | NA |
| SOX11 | Ab59776; Abcam | 5% milk; 1:2000 | NA | NA |
| SMAD4 | Sc7154; Santa Cruz | 3% BSA; 1:3000 | NA | NA |
| FZD4 | Ab83042; Abcam | 5% milk; 1:500 | NA | NA |
| FZD7 | Ab51049; Abcam | 3% BSA; 1:500 | NA | NA |
| AKT1/2 | Ab8933; Abcam | 3% BSA; 1:1000 | NA | NA |
| Cyclin D1 | Ab16663; Abcam | 3% BSA; 1:200 | NA | TE Buffer (10mMTriss 1mM EDTA), 3% BSA, 1:50 |
| α tubulin | T5168 Sigma | 3% BSA; 1:8000 | NA | NA |
