# Supplementary material for: A Unique Combination of Male Germ Cell miRNAs Coordinates Gonocyte Differentiation
Source: PLoS One. 2012 Apr 20;7(4):e35553. doi: 10.1371/journal.pone.0035553 (PMC3334999; doi:10.1371/journal.pone.0035553)
Supplement: Table S4 — qPCR primer sequences and annealing temperatures. Forward and reverse primer sequences (Sigma) used to selectively amplify the genes of interest with their associated annealing temperatures. For the miRNA sequences capital letters indicate the location of locked nucleic acids used to raise the melting temperature of the primer (GeneWorks, Hindmarsh, SA, Australia). (DOC) [file pone.0035553.s006.doc]

Table S2: qPCR primer sequences and annealing temperatures.

Forward and reverse primer sequences (Sigma) used to selectively amplify the genes of interest with their associated annealing temperatures. For the miRNA sequences capital letters indicate the location of locked nucleic acids used to raise the melting temperature of the primer (GeneWorks, Hindmarsh, SA, Australia).

| Gene | Forward primer (5’-3’) | Reverse primer (5’-3’) | Annealing temperature ^o^C |
| --- | --- | --- | --- |
| AKT1 | actcattccagacccacgac | gtccagggcagacacaatct | 63 |
| BMPR1a | aggggtcgttacaaccgtga | accaacctgccgaaccatct | 65 |
| Cyclin D1 | tgtgctgcgaagtggagacc | agttcatggccagcgggaag | 65 |
| Cyclophilin | cgtctccttcgagctgttt | acctggcacatgaatcct | 64.9 |
| FZD4 | ctgacaactttcacgccgct | gggccaggcaaacccaaatt | 62.9 |
| FZD7 | gcttcggattccgtgcaact | tgctccgccttctctccttg | 65 |
| Nanog | cgttcccagaattcgatgctt | ttttcagaaatcccttccctc | 58.3 |
| Ngn3 | gcttctcatcggtaccct | gtgatggatggtcggagg | 65 |
| Oct3/4 | accttcaggagatatgcaaatcg | ttctcaatgctagttcgctttctct | 58.3 |
| PLZF | gcacactcaagagccaca | ccgttgtgtgttctcaggtgc | 65 |
| PTEN | acagccatcatcaaagagatcgt | tcaagtctttctgcaggaaatccca | 62.9 |
| Smad4 | tatgcccgtctgtggaggtg | tcagtgggtaaggacggctg | 65 |
| Sox11 | ggactttgcaacttgccgga | atgaactcgccctcctcgg | 62.9 |
| U6 | tcgcttcggcagcacatatact | cgcttcacgaatttgcgtgtca | 50 |
| Mmu-miR-463* | aCtCcatttgtttt | gtaaaacgacggccagtatgatgga | 50 |
| Mmu-miR-136 | aCtCcatttgtttt | gtaaaacgacggccagtccatcatc | 50 |
| Mmu-miR-743a | gaaagacaccaagc | gtaaaacgacggccagttctactca | 50 |
| Mmu-miR-293 | agtgccgcagagt | gtaaaacgacggccagtacactaca | 50 |
| Mmu-miR-291a-5p | catcaaagtggagg | gtaaaacgacggccagtagagaggg | 50 |
| Mmu-miR-294* | aCtcaaaatggagg | gtaaaacgacggccagtagataggg | 50 |
| Mmu-miR-290-5p | aCtcaaactatggg | gtaaaacgacggccagtaaagtgcc | 50 |
| M13F |  | gtaaaacgacggccagt |  |
